# High-Yield Expression of Heterologous [FeFe] Hydrogenases in *Escherichia coli*

**DOI:** 10.1371/journal.pone.0015491

**Published:** 2010-11-24

**Authors:** Jon M. Kuchenreuther, Celestine S. Grady-Smith, Alyssa S. Bingham, Simon J. George, Stephen P. Cramer, James R. Swartz

**Affiliations:** 1 Department of Chemical Engineering, Stanford University, Stanford, California, United States of America; 2 Department of Applied Science, University of California Davis, Davis, California, United States of America; 3 Physical Biosciences Division, Lawrence Berkeley National Laboratory, Berkeley, California, United States of America; 4 Department of Bioengineering, Stanford University, Stanford, California, United States of America; University of Delhi, India

## Abstract

**Background:**

The realization of hydrogenase-based technologies for renewable H_2_ production is presently limited by the need for scalable and high-yielding methods to supply active hydrogenases and their required maturases.

**Principal Findings:**

In this report, we describe an improved *Escherichia coli*-based expression system capable of producing 8–30 mg of purified, active [FeFe] hydrogenase per liter of culture, volumetric yields at least 10-fold greater than previously reported. Specifically, we overcame two problems associated with other *in vivo* production methods: low protein yields and ineffective hydrogenase maturation. The addition of glucose to the growth medium enhances anaerobic metabolism and growth during hydrogenase expression, which substantially increases total yields. Also, we combine iron and cysteine supplementation with the use of an *E. coli* strain upregulated for iron-sulfur cluster protein accumulation. These measures dramatically improve *in vivo* hydrogenase activation. Two hydrogenases, HydA1 from *Chlamydomonas reinhardtii* and HydA (CpI) from *Clostridium pasteurianum*, were produced with this improved system and subsequently purified. Biophysical characterization and FTIR spectroscopic analysis of these enzymes indicate that they harbor the H-cluster and catalyze H_2_ evolution with rates comparable to those of enzymes isolated from their respective native organisms.

**Significance:**

The production system we describe will facilitate basic hydrogenase investigations as well as the development of new technologies that utilize these prolific H_2_-producing enzymes. These methods can also be extended for producing and studying a variety of oxygen-sensitive iron-sulfur proteins as well as other proteins requiring anoxic environments.

## Introduction

Molecular hydrogen (H_2_) is an important feedstock for the synthesis of chemicals and fertilizers, and it also has great potential as a clean carrier of energy for renewable fuel technologies. However, conventional means for industrial-scale H_2_ production such as steam reformation of natural gas fall short of the environmental criteria now needed for sustainable fuels and chemicals [1]. The use of hydrogenase enzymes offers a promising alternative to traditional H_2_ production technologies.

Hydrogenases catalyze the redox interconversion of protons and hydrogen gas (2H^+^ +2e^−^ 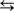 H_2_) using unique transition metal cofactors by which the enzymes are classified. Since [FeFe] hydrogenases more rapidly and preferentially evolve H_2_ than [NiFe] hydrogenases [2], [3], they are more desirable for H_2_ production technologies. Unfortunately, these prolific H_2_ producing enzymes are also easily inactivated by oxygen.

The [FeFe] hydrogenase active site cofactor, termed the H-cluster, is a complex iron-sulfur cluster that is stabilized by carbon monoxide and cyanide ligands as well as a dithiol bridging molecule [4], [5], [6]. H-cluster assembly and active hydrogenase expression require at least three accessory proteins called the HydE, HydF, and HydG maturases [7]. Recent investigations have provided valuable insights regarding H-cluster synthesis. For example, tyrosine was first implicated as an essential substrate for hydrogenase activation [8], and subsequent work revealed that this amino acid is likely the source for the carbon monoxide and cyanide ligands [9], [10]. A cationically charged channel has also been identified through which the H-cluster cofactor is inserted into the hydrogenase apoenzyme [11], [12], possibly from the HydF maturase [13], [14]. Despite these advances, however, the concerted functionality of the maturases and complete H-cluster biosynthetic pathway have yet to be elucidated [15]. In addition to the challenges imposed by the complexity of these enzymes and the maturation process, most work with hydrogenases and their maturases must be done in strictly anaerobic environments. The reduced nature of the H-cluster and accessory iron-sulfur clusters (ISCs) makes them susceptible to damage by O_2_ oxidation.

Research groups have overcome these challenges and have used hydrogenases for energy conversion at the laboratory scale in several different applications. Protein complexes attached to solid-state devices have evolved H_2_ using electrons activated by light [16], [17]. Photoelectrochemical fuel cells with hydrogenases adsorbed to cathodic carbon electrodes have preferentially evolved H_2_ and perform similarly to fuel cells that use platinum catalysts [18]. Also, synthetic metabolic pathways assembled with purified enzymes have converted sugars to H_2_ and CO_2_ at high yields [19], [20]. Despite the successful development of these hydrogenase technologies, their commercial realization and sustainability will require large quantities of active protein. Various microbial systems have been engineered for producing native and heterologous [FeFe] hydrogenases [21], [22], [23], [24], [25], [26], [27], but active enzyme yields are generally less than 1 mg·L^−1^ of culture. Also, few recombinant DNA tools exist for effective overexpression of proteins in organisms that naturally harbor [FeFe] hydrogenases.

*Escherichia coli* has several advantages that make it desirable for hydrogenase production. The bacterium does not contain a native [FeFe] hydrogenase that needs to be knocked out to simplify analytical measurements, it is capable of anaerobic respiration, and heterologous expression techniques for this microbe are well-established. Active hydrogenase production using *E. coli* systems has been demonstrated, with total yields comparable to the best reported. However, specific activities of purified hydrogenases from these systems are significantly lower than activities of hydrogenases isolated from their native hosts, likely because of incomplete enzyme maturation [22], [24], [25].

In this report, we describe the high-yield production of active [FeFe] hydrogenases using the maturases native to *Shewanella oneidensis* along with *E. coli* BL21(DE3) *ΔiscR*, a strain previously engineered for improved synthesis of iron-sulfur (Fe–S) proteins [22]. Following expression with our optimized protocol, both the *C. reinhardtii* HydA1 and *C. pasteurianum* CpI hydrogenases were isolated by *Strep*–Tactin affinity chromatography and characterized using activity assays and Fourier transform infrared (FTIR) spectroscopy.

## Results

### Recombinant Protein Expression Concurrent with Anaerobic Metabolism and Growth

**Figure 1:**
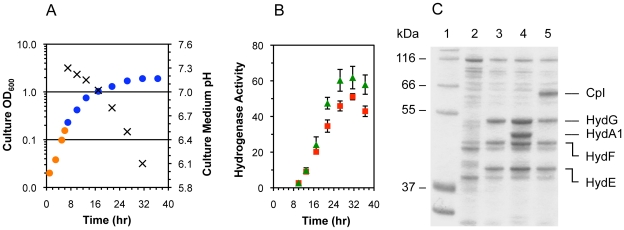
*E. coli* growth and anaerobic expression of heterologous active [FeFe] hydrogenases. All data are for cultures of *E. coli* strain BL21(DE3) *ΔiscR*, and both iron and cysteine were included in the growth medium. (Fig. 1A) Optical density at 600 nm (shown on a logarithmic scale) of cultures during aerobic (orange circles) and anaerobic (blue circles) growth phases for cells containing the pACYCDuet-1–*hydGX*–*hydEF* and pET-21(b) shydA1*–Strep-tag II plasmids. The pH of culture media (×) was also measured. Data for cultures with cells containing the pET-21(b) *shydA–Strep-tag II* plasmid instead of pET-21(b) shydA1*–Strep-tag II were similar and are not shown. (Fig. 1B) Cell lysate-based hydrogenase activities (µmol MV reduced·min^−1^·mg^−1^ total protein) for active CpI (red squares) and HydA1 hydrogenase (green triangles) were determined using the methyl viologen reduction assay. Data are the average for n = 3 cultures examined ± standard deviations. (Fig. 1C) SDS-PAGE analysis for the soluble fractions of final cell lysates after the anoxic co-expression of HydA1 or CpI and the HydE, HydF, and HydG maturases: (Lane 1) the molecular weight markers are from the Mark12^TM^ protein ladder (Invitrogen); (Lane 2) soluble cell lysate protein content for *E. coli* strain BL21(DE3) *ΔiscR* following expression of no heterologous proteins from recombinant DNA plasmids; (Lane 3) co-expression of only the HydE, HydF, and HydG maturases; (Lane 4) co-expression of HydE, HydF, HydG, and HydA1–*Strep*-tag II; and (Lane 5) co-expression of HydE, HydF, HydG, and CpI–*Strep*-tag II.

Co-expression of the [FeFe] hydrogenases and the maturases was induced under strictly anoxic conditions at an optical density (OD_600_) of ∼0.4. To facilitate anaerobic metabolism, glucose (0.5% w/v) and the electron acceptor fumarate (25 mM) were added to the complex growth medium. Aerobic growth rates were exponential (0.45 hr^−1^), while anaerobic growth rates were linear and eventually ceased after 24 hr at final OD_600_ measurements ranging from 1.5 to 3.0 (Fig. 1A). Substrate limitations and acetate accumulation may have contributed to the slowed anoxic growth. Without glucose addition, the culture density did not increase during the anaerobic incubation period. This lack of growth resulted in a lower cell concentration, which thus decreased the total amount of hydrogenase produced per culture volume.

Active hydrogenase levels were determined in samples taken during the anaerobic growth period by measuring the methyl viologen reduction activities of cell lysates. This assay for hydrogenase-catalyzed H_2_ uptake enabled us to identify the conditions for optimal active enzyme production. For both the HydA1 and CpI hydrogenases, maximal H_2_ uptake activities were observed after 20–24 hr of anaerobic incubation (Fig. 1B), and the four heterologous proteins had accumulated to become abundant proteins, based on SDS-PAGE analysis (Fig. 1C). Increased rates of cell death accompanied by protein degradation could explain the modest decrease in activity after 24 hr, as anoxic growth appeared to cease at this time. Only minimal amounts of methyl viologen reduction (less than 1% of the maximal activities) were observed when either the hydrogenase alone or only the three maturases were expressed. Thus, methyl viologen reduction could be specifically attributed to active [FeFe] hydrogenase in the cell lysates and not the maturases or native *E. coli* [NiFe] hydrogenases.

**Figure 2:**
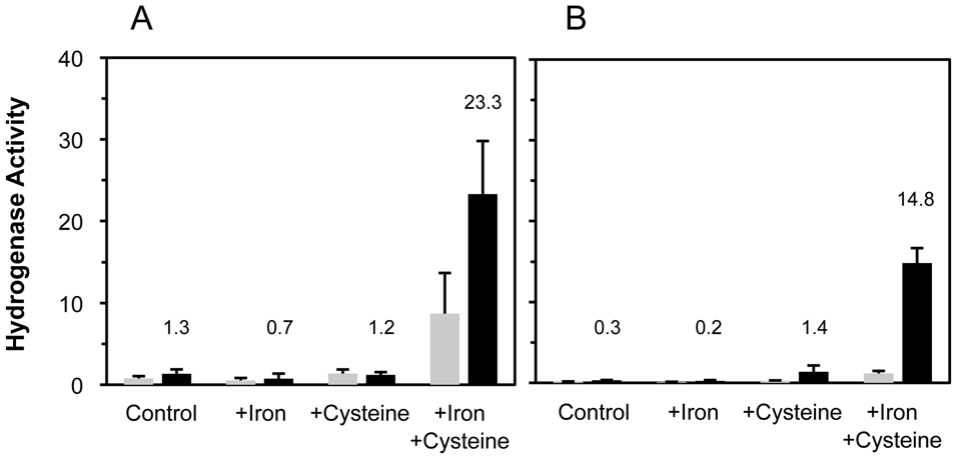
Effects of iron and cysteine supplementation as well as the *iscR* deletion on active hydrogenase expression. Iron (2 mM ferric ammonium citrate) and cysteine (2 mM) were added to cultures as indicated. Methyl viologen reducing activities (µmol MV reduced·min^−1^·mg^−1^ total protein) of active [FeFe] hydrogenase in cell lysates from *E. coli* strains BL21(DE3) (gray bars) and BL21(DE3) *ΔiscR* (black bars). Hydrogenase activities were measured after 16–18 hrs of anaerobic HydA1 expression (A) and CpI expression (B). Hydrogenase activities for the *ΔiscR*-derived samples are indicated above the respective columns, and all activities are the average for n = 2 cultures ± standard deviations.

[FeFe] hydrogenases and each of the three maturases require ISCs in order to function [28], [29], and recombinant overexpression of the four Fe–S proteins likely creates increased demand for ISC assembly. We therefore investigated the benefits of supplementing potentially limiting substrates as well as using the mutant *ΔiscR* strain engineered for improved production of proteins harboring ISCs [22]. Expression with the *ΔiscR* strain improved active hydrogenase production 2–10 fold, with a greater benefit for CpI production (Fig. 2). Addition of both iron (2 mM ferric ammonium citrate) and cysteine (2 mM) to the culture medium resulted in a further 5–10 fold increase in hydrogenase activity. Neither additive individually improved hydrogenase activation to the same level, although cysteine supplementation led to a moderate improvement in active CpI expression when using the *ΔiscR* strain. The cooperative effect of iron and cysteine suggests that both substrates are limiting when overexpressing proteins harboring ISCs. We also tested the addition of other relevant hydrogenase maturation substrates to the medium, along with the iron and cysteine. These substrates were *S*-adenosyl methionine (SAM; 2 mM), tyrosine (2 mM), and methionine (2 mM). Adding these individually or in combination did not increase hydrogenase activities (data not shown). Overall, expression with the *ΔiscR* strain receiving iron and cysteine supplementation enhanced HydA1 activities 25-fold and CpI activities 100-fold.

### Biophysical Characterization of Purified [FeFe] Hydrogenases

Based on the results presented in Figure 2, HydA1 and CpI were expressed using the conditions identified for maximal hydrogenase activities and were subsequently isolated to high purity with *Strep*-Tactin affinity chromatography. The elution fractions containing active hydrogenase were identified using the methyl viologen reduction assay as well as by SDS-PAGE analysis. Generally, 70–90% of the total activity present in the cell lysates was recovered in the elution fractions. The purification yields for HydA1 and CpI were 30 mg·L^−1^ of culture and 8 mg·L^−1^ of culture, respectively (Table 1).

**Table 1:** Biophysical characterization of purified [FeFe] hydrogenases produced in *Escherichia coli*.

	HydA1	CpI
**H_2_ Uptake (MV)** †	251±49	476±39
µmol H_2_·min^−1^·mg^−1^		
**H_2_ Evolution (MV)** †	641±88	1087±146
µmol H_2_·min^−1^·mg^−1^		
**H_2_ Evolution (PetF)** †	41±4	90±10
µmol H_2_·min^−1^·mg^−1^		
**Iron Content**	4.5±0.2	13.2±1.3
Fe atoms·peptide^−1^		
**Purification Yield**	30±11	7.9±0.8
mg·L^−1^ of culture		

Data are the average from n = 3–6 cultures examined ± standard deviations.

† Specific activities of purified hydrogenases were determined using methyl viologen (MV) or PetF ferredoxin as the electron donating or accepting substrates.

Both specific activities and protein-bound iron content were determined for the purified HydA1 and CpI enzymes, and the results are summarized in Table 1. H_2_ uptake rates as well as H_2_ evolution rates are higher for CpI in all cases, and both HydA1 and CpI contained ∼70% of the maximum amount of iron (6 and 20 iron atoms are expected for these hydrogenases, respectively). We also used the purified *Synechocystis* [2Fe–2S] ferredoxin PetF (50 µM) instead of methyl viologen as the electron donating substrate. Since ferredoxin proteins are the native electron transport substrates for [FeFe] hydrogenases, the catalytic rates when using PetF are more relevant for future design efforts to engineer microbial H_2_ production systems. We observed a K_m_ of 20 µM for PetF with CpI when using sodium dithionite (DTH) as the source of electrons for ferredoxin reduction. Reduced PetF, however, supported significantly lower H_2_ evolution rates compared to methyl viologen. Lower catalytic rates when using a [2Fe–2S] ferredoxin have been previously observed when compared to rates observed when a [4Fe–4S] ferredoxin was used [30].

### FTIR Spectroscopic Analysis of Isolated [FeFe] Hydrogenases

**Figure 3:**
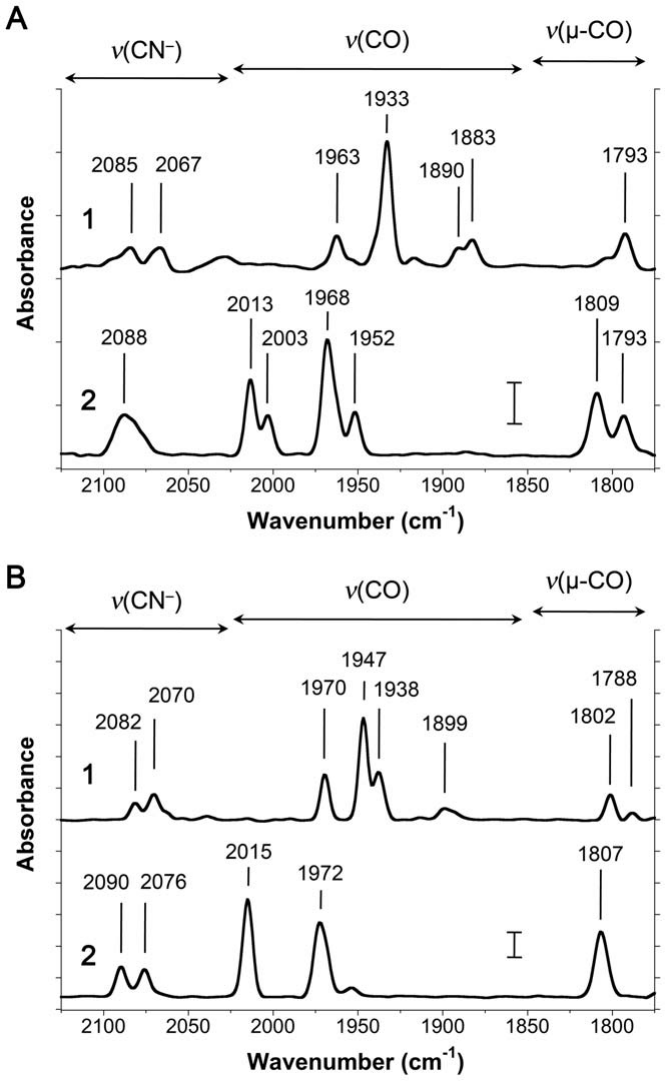
Fourier transform infrared spectra of heterologous [FeFe] hydrogenases produced in *E. coli*. Infrared spectra are for 100–200 µM of the (A) HydA1 and (B) CpI hydrogenases. Both enzymes were examined [**1**] in their as-isolated state as well as [**2**] following treatment with exogenous CO. Vibrational energies (in cm^−1^) for the H-cluster CO and CN^−^ ligands are indicated in each spectrum. The wavenumber ranges for terminal CN^−^ (*ν*(CN^−^)), terminal CO (*ν*(CO)), and bridging CO (*ν*(µ-CO)) vibrational modes are shown above the spectra. Scale bars shown at 1870 cm^−1^ represent a difference of 0.5 milliabsorbance units.

FTIR spectroscopy was used to analyze the purified HydA1 (Fig. 3A) and CpI hydrogenases (Fig. 3B) in both the as-isolated state as well as following treatment with exogenous CO. The spectra for both as-isolated hydrogenases clearly show peaks representing CN^−^ and CO vibrational stretches, indicating the presence of fully assembled H-clusters. Based on previous reports for each of these enzymes [31], [32] as well as other [FeFe] hydrogenases [33], these spectra also indicate that the as-isolated hydrogenases have a mixture of H-clusters in both the oxidized (H_ox_) and reduced (H_red_) states. The presence of DTH in the elution buffer was essential to prevent hydrogenase inactivation during purification, and this additive was likely the cause for the mixture of H-cluster redox states. The CO inhibition studies confirmed the presence of the H-cluster cofactors, as the CO and CN^−^ vibrational modes shifted as expected after exogenous CO binding to the H-cluster.

## Discussion

By implementing several changes for heterologous [FeFe] hydrogenase expression, we achieved HydA1 and CpI yields more than 10-fold higher than previously reported for these enzymes (Table 2). Enabling concurrent anaerobic cell growth and T7 RNA polymerase induction was essential for increased heterologous protein production. In the absence of anoxic cell growth (i.e. incubation without glucose added to the medium), anaerobic hydrogenase and maturase accumulation levels were noticeably lower, as indicated by both hydrogenase activity assays and SDS-PAGE analysis (data not shown). This decrease in protein expression could be expected since translation is energy intensive due to the high entropic demands, and the rates of ATP synthesis are dramatically reduced under non-respiring conditions.

**Table 2:** Comparing this work to previous reports for the production of the *C. reinhardtii* HydA1 hydrogenase.

Microbial host	*C.r.* HydA1 Variant	Specific Activity^1^	Purification Yield^2^	Iron Content^3^	Ref.
*C. reinhardtii*	HydA1	730±146	0.04	NR	[23]
	HydA1	935	0.001	3.9±0.3	[35]
*S. oneidensis*	*Strep-*tag II–HydA1	740±56	0.5	6.1±0.1	[26]
*C. acetobutylicum*	HydA1–*Strep-*tag II	625	1.0	NR	[27]
	HydA1–*Strep-*tag II	760	0.1	NR	[37]
*E. coli*	HydA1–*Strep-*tag II	150	1.0	NR	[24]
***E. coli***	**HydA1–** ***Strep-*** **tag II**	**641±88**	**30±11**	**4.5±0.2**	**This Work**

1 Specific activities for H_2_ production rates are expressed in units of µmol H_2_·min^−1^·mg^−1^ of HydA1.

2 Purification yields are mg of HydA1 isolated per liter of cell culture.

3 Iron content is expressed in units of Fe atoms per HydA1 peptide; NR, not reported.

Along with increased yields, the purified hydrogenases are also highly active and contain a properly assembled H-cluster based on *in vitro* enzymatic activities as well as FTIR spectroscopic analyses. Previous studies using *E. coli* as an expression host for heterologous [FeFe] hydrogenase production reported specific activities much lower than those measured for the same protein purified from its native host (Table 2), likely due to incomplete hydrogenase maturation as well as possible loss of activity during purification [22], [24], [25]. The CpI and HydA1 enzymes produced with our optimized system have specific activities similar to those of the respective wild-type enzymes isolated from their native hosts [23], [34]. The HydA1 hydrogenase isolated from *C. reinhardtii* was shown to evolve H_2_ with rates 650–950 µmol H_2_·min^−1^·mg^−1^ [23], [35], while CpI from *C. pasteurianum* was shown to evolve H_2_ with rates of 1100–5500 µmol H_2_·min^−1^·mg^−1^, depending on the assay conditions [2], [6], [21], [34]. While it appears that all HydA1 enzymes were active, we estimate that 20–40% of the CpI enzymes were not. Considering that CpI requires additional accessory iron-sulfur clusters, it is possible that this enzyme is more difficult to mature compared to HydA1. The iron contents for both hydrogenases that we examined were measured to be ∼70% of that expected, which is consistent with the observation of lower-than-expected specific activity. Alternatively, experimental error associated with measuring iron quantities and protein concentration could also account for the lower-than-expected iron content, and some of the hydrogenases may have been inactivated during the purification process. The latter of these theories agrees with the 70% recovery of total hydrogenase activity during purification, which is further discussed below.

Like the [FeFe] hydrogenases, HydE, HydF, and HydG also require ISCs. Thus, the benefits of the engineered *ΔiscR* strain along with iron and cysteine supplementation could be expected since not one, but four Fe–S proteins must be overexpressed [22]. The more pronounced benefit of the *ΔiscR* strain for active CpI expression (Fig. 2B) might be explained by the enzyme's requirement for N-terminal ISCs. Unlike the algal HydA1 hydrogenase, CpI has three [4Fe–4S] clusters and one [2Fe–2S] cluster that participate in electron transfer to the H-cluster cofactor [6]. As transcription of the *E. coli isc* operon is deregulated in the *ΔiscR* strain [36], higher expression of the corresponding native ISC proteins likely enhances the assembly, installation, and/or repair of these four accessory ISCs. The benefit of cysteine supplementation for active CpI expression in the *ΔiscR* strain further supports this hypothesis, as increased *in vivo* levels of the cysteine desulfurase IscS may improve cysteine utilization for ISC biosynthesis.

With our methods and a single purification step, we obtained greater than 70% recovery of the *in vivo* hydrogenase activity. During our experimentation, we identified several factors that affected the overall efficacy of the purification process such as the necessity for DTH during protein purification. When buffers did not contain fresh DTH, both HydA1 and CpI more rapidly deactivated, in agreement with previously reported observations [25], [35]. We also used a commercial lysis buffer (BugBuster Master Mix) to produce the lysates as this approach is simpler (given the anaerobic requirements) than alternative methods such as sonication and homogenization. High-yield expression was also beneficial for efficient recovery, since hydrogenase concentrations in the lysates (estimated to be 5–25 µM) are then higher than the K_d_ for *Strep*-tag II:*Strep*-Tactin adsorption (1 µM). The affinity tag location was another important factor as observed in other studies [37]. While the presence of a C-terminal affinity tag did not seem to negatively affect the solubility or catalytic properties of the hydrogenases, we could not produce soluble CpI with an N-terminal affinity tag. Moreover, HydA1 with an N-terminal affinity tag had a 50% lower specific activity, as indicated by both methyl viologen-based activity assays (data not shown). Traditional methods for isolating HydA1 without an affinity tag have combined multiple purification steps, and the majority of the hydrogenase activity (80–90%) was lost during the procedures [23], [35]. Also, immobilized metal ion affinity chromatography (IMAC) could cause detrimental interactions between protein metal clusters and the resin [38], and high salt concentrations are generally used to recover the bound protein. *Strep*-Tactin affinity chromatography may also be more favorable for purifying metalloproteins compared to multi-step chromatography or IMAC. The *Strep*-Tactin approach involves a single chromatography step for efficient recovery of pure protein. Moreover, since buffer exchanges are not required to deplete high salt concentrations, the preparation of concentrated hydrogenase samples for FTIR spectroscopic analysis is simpler and less time consuming.

The production of two structurally different [FeFe] hydrogenases using heterologous maturases illustrates the versatility of this expression system. Infrared spectroscopic data confirm the presence of CO and CN^–^ ligands, indicating that both HydA1 and CpI contain an intact H-cluster identical to that of the protein produced in the native organisms and assembled by the native maturases. Despite the evolutionary diversity of [FeFe] hydrogenases, H-cluster biosynthesis and hydrogenase maturation appear to be highly conserved. Our results also underscore the modularity of the microbial world and the potential for dramatic evolutionary change through DNA exchange. It thus seems likely that the HydE, HydF, and HydG maturases from *S. oneidensis* could also activate other [FeFe] hydrogenases of interest (e.g. hydrogenases from *Thermatoga maritima* [39] and *Desulfovibrio vulgaris* [25]). One advantage of using the *S. oneidensis* maturases is the similarity between *Shewanella and Escherichia*. For example, high yields and soluble expression of HydE, HydF, and HydG were observed (Fig. 1C), even without codon optimizing the maturase genes.

The effectiveness of *E. coli* for inducible expression of heterologous proteins along with the variety of commercial recombinant DNA expression tools make this organism more desirable than others (e.g. *Clostridia*) for the large-scale production of hydrogenases for a variety of applications. In this work, we illustrate this advantage via the facile production of hydrogenases for IR spectroscopic analysis. Such analytical techniques generally require large quantities of hydrogenase per sample (>500 µg) at concentrations greater than 5 mg·mL^−1^. With our system, sufficient quantities of HydA1 and CpI hydrogenase for multiple IR spectroscopic measurements can be obtained from a single 250 mL culture. In comparison, isolation of HydA1 from its native host requires 8 L of culture and multiple purification steps to produce enough hydrogenase for one IR spectroscopic measurement [23].

### Conclusions

The commercialization of technologies that use [FeFe] hydrogenases will most certainly require economical approaches for producing these complex oxygen-sensitive enzymes. Furthermore, much remains unknown about H-cluster biosynthesis and the hydrogenase maturation process. We provide a new, effective protocol for producing these enzymes to greatly facilitate both technology development and hydrogenase biochemistry research. The methods we describe could also be extended for producing other enzymes associated with anaerobic metabolism such as [NiFe] hydrogenases and nitrogenases.

## Materials and Methods

### [FeFe] Hydrogenase and Maturase Expression Constructs

The *C. reinhardtii hydA1* and *C. pasteurianum hydA* genes were used for expression of the HydA1 and CpI [FeFe] hydrogenases, respectively. Both genes were previously codon-optimized for expression in *E. coli* [40]. The coding sequencing for a C-terminal *Strep*-tag II® extension (IBA GmbH) with a two residue linker (5′-SAWSHPQFEK-3′) was added by PCR amplifying the hydrogenase genes from the plasmids pY71 shydA1* [8] and pK7 *shydA* [40]. PCR products were then cloned into the pET-21(b) expression vector (Novagen). The plasmid pACYCDuet-1–*hydGX*–*hydEF* [8] was used for expression of the *S. oneidensis* [FeFe] hydrogenase maturases HydE, HydF, and HydG. Multiple cloning sites I and II contain the *hydGX* and *hydEF* nucleotide sequences, respectively. The *hydX* sequence (Accession code AAN56899) is a part of the *S. oneidensis* [FeFe] hydrogenase operon and encodes a soluble protein with no identified functions. The *petF* gene from *Synechocystis sp.* PCC 6803 was PCR amplified from the pK7 expression vector and cloned into the pET-21(b) plasmid [41]. All expression constructs were confirmed by DNA sequencing and transformed into the *E. coli* strains BL21(DE3) (Novagen) and BL21(DE3) *ΔiscR* by selection with the appropriate antibiotics.

### Recombinant Expression and Purification of Active Hydrogenases

*E. coli* strains BL21(DE3) and BL21(DE3) *ΔiscR* contained the pACYCDuet-1–*hydGX*–*hydEF* plasmid and one of the two pET-21(b) hydrogenase plasmids. Cells were grown in LB Miller growth medium supplemented with kanamycin (40 mg·L^−1^; only when using the *ΔiscR* strain), chloramphenicol (25 mg·L^−1^), ampicillin (100 mg·L^−1^), 0.5% w/v glucose (∼25 mM), and 100 mM MOPS/NaOH (final pH of medium was 7.4). The *ΔiscR* strain contains a chromosomal substitution of the *iscR* gene with another gene conferring resistance to kanamycin [22]. 10–50 mL cultures were grown for investigating the effects of cell strains and substrates, while 50–250 mL cultures were grown for hydrogenase purification work. Initially, all cultures were grown aerobically at 25°C until an OD_600_ of 0.3–0.5. They were then moved into an anaerobic glove box (Coy Laboratory Products) containing 98% N_2_ and 2% H_2_ prior to IPTG-based T7 RNA polymerase induction and heterologous protein expression. While ferric ammonium citrate (2 mM) was added to the growth medium prior to inoculation, both cysteine (2 mM) and sodium fumarate (25 mM) were added with IPTG (0.5 mM) within the anaerobic glove box. Cultures were sealed and incubated at 25°C for 16–24 hours following induction.

For investigating media formulations and protein expression by different strains, cells from 1 mL of culture were pelleted at 4,000×g and resuspended in 100 µL of anaerobic BugBuster® Master Mix lysis solution (Novagen) containing an additional 25 mM Tris/HCl (pH 8.0), 25 mM KCl, 3 mM sodium dithionite (DTH), 1 mM dithiothreitol (DTT), 2% v/v glycerol, 0.1% v/v Tween 20, 0.2 mM phenylmethylsulfonyl fluoride (PMSF), and 2 µM resazurin as an oxygen indicator. After cell lysis (incubation at 25°C for 20 min), lysates were clarified by centrifugation at 14,000×g. Hydrogenase activities in cell lysates were measured using the methyl viologen reduction assay described below. Total protein content of lysates was determined using a commercial assay (Bio-Rad) based on the method of Bradford [42], and the extent of heterologous protein expression was visualized using polyacrylamide gel electrophoresis with SDS-PAGE gels (Invitrogen).

Hydrogenase purifications were carried out while maintaining strict anaerobic conditions. After centrifugation and lysis as described above, approximately 1 mL of *Strep*-Tactin® Superflow® high capacity resin (IBA GmbH) was used per 50 mL of cell culture for purification. Wash and elution buffers contained the above lysis buffer additives excluding the BugBuster Master Mix and PMSF, and active hydrogenase was eluted with 2.5 mM D-desthiobiotin. Elution fractions were evaluated for active hydrogenase using the methyl viologen reduction assay, and fractions with high activity were pooled. Protein concentrations were measured with the Bradford assay, and hydrogenase iron content was measured using a ferrozine-based colorimetric assay [43]. Hydrogenase samples for IR spectroscopic studies were anaerobically concentrated to ∼100 µM using a 10 mL stirred cell and a 5 kD MWCO membrane (Amicon). Hydrogenase samples were not frozen prior to characterization and spectroscopic analysis.

### Hydrogenase Activity Assays

Hydrogenase activities were measured at 37°C in both the H_2_ consumption and H_2_ evolution directions using methods previously described [40]. Generally, 1–25 ng of hydrogenase was tested. H_2_ uptake was quantified with a methyl viologen reduction assay. 200 µL assay solutions contained 50 mM Tris/HCl (pH 8.0) and 2 mM methyl viologen. Absorbance was measured at 578 nm for 1–2 min following addition of lysate or purified hydrogenase. Methyl viologen reduction rates were adjusted by subtracting background activities, which were generally less than 1% of the hydrogenase activities. An extinction coefficient for reduced methyl viologen of 9.78 mM^−1^·cm^−1^ was used to calculate H_2_ oxidation rates, in which 2 moles of methyl viologen are reduced per mole of H_2_ consumed. Hydrogen production was quantified using DTH-reduced methyl viologen (5 mM) or the ferredoxin PetF from *Synechocystis* (50 µM) as the electron donating substrate. 9.5 mL glass vials contained 1 mL of 100 mM MOPS/KOH buffer (pH 6.8), 100 mM KCl, 25 mM DTH, and either methyl viologen or ferredoxin. Upon hydrogenase addition, vials were sealed and the headspace was sparged with 100% N_2_ for 2 min. H_2_ quantities in the headspace were then quantified after 15–30 min of incubation using a ShinCarbon ST 100/120 mesh column (Resteck) with a Hewlett Packard 6890 gas chromatograph (Hewlett Packard). For PetF ferredoxin production, the *petF* gene from *Synechocystis sp.* PCC 6803 was first cloned from the pK7 plasmid [40] into the pET-21(b) vector, and subsequently transformed into BL21(DE3) *ΔiscR*. Both PetF expression and purification using ammonium sulfate precipitation followed by anion exchange chromatography were carried out as previously described [41], [44].

### Fourier Transform IR Spectroscopy

Infrared spectra were measured using a Bruker IFS/66s FTIR spectrometer interfaced to a home-built stopped-flow drive system. The IR sample cuvette and drive system were maintained inside an anaerobic chamber (O_2_<1.1 ppm) as previously described [45]. The sample cuvette was maintained at 25°C with a calibrated path length of 47.6 µm. For IR spectroscopic measurements, one drive syringe contained the protein sample. A second syringe contained either the elution buffer without any protein or buffer saturated with exogenous carbon monoxide. Spectra were measured at 4 cm^−1^ resolution from 1000 sample scans, and the average spectrum was improved with a background correction.
